# Sleep disturbances in Taiwanese patients with Parkinson's disease

**DOI:** 10.1002/brb3.806

**Published:** 2017-09-21

**Authors:** Yi‐Ying Lin, Rou‐Shayn Chen, Chin‐Song Lu, Ying‐Zu Huang, Yi‐Hsin Weng, Tu‐Hsueh Yeh, Wey‐Yil Lin, June Hung

**Affiliations:** ^1^ Department of Neurology LinKou Chang Gung Memorial Hospital Taoyuan Taiwan

**Keywords:** excessive daytime sleepiness, Parkinson's disease, Parkinson's Disease Sleep Scale, Pittsburgh Sleep Quality Index, sleep disturbance, Taiwanese

## Abstract

**Introduction:**

Sleep disturbance is a common nonmotor symptom of Parkinson's disease (PD) and strongly affects patients’ quality of life. The relationship between excessive daytime sleepiness (EDS) and nighttime problems remains uncertain. Arguments persist regarding the risk factors for sleep disturbance among patients with PD. Furthermore, the prevalence of EDS appears to be lower in Asian countries. Herein, we conducted the study to describe the characteristics of sleep problems in a sample of Taiwanese PD patients and delineate the difference with reported sleep disturbances in Caucasian PD patients from the literature.

**Methods:**

Patients with PD were recruited from the outpatient clinic of a tertiary medical center and were evaluated using standardized assessment protocols, including the Parkinson's Disease Sleep Scale (PDSS), the Pittsburgh Sleep Quality Index (PSQI), Epworth Sleepiness Scale (ESS), and 39‐Item Parkinson's Disease Questionnaire (PDQ‐39).

**Results:**

A total of 225 patients with PD were recruited. The mean age of patients with PD was 65.7 years old and the mean disease duration was 8.18 years. Among the patients, 53.8% were defined as poor sleepers (PSQI > 5) and 26.3% had EDS. Seventy‐one percent of the poor sleepers used hypnotic medications. The poor sleepers were worse in the scores of Unified Parkinson's Disease Rating Scale (UPDRS), PDSS, and the PDQ‐39, and received higher levodopa daily dosage. A PDSS score of <126 indicate that a patient with PD was a poor sleeper. EDS was positively correlated with advanced Hoehn and Yahr stages and use of dopamine agonists but not with levodopa daily dosage and levodopa equivalent daily dosage.

**Conclusions:**

Taiwanese patients with PD had a lower prevalence of EDS compared with the literatures reported in Caucasian patients. We identify and suggest that PDSS total score, rather than subcategory items, should be used to predict poor sleep among patients with PD.

## 1 INTRODUCTION

Sleep disturbance is common among patients with Parkinson's disease (PD). Approximately 37%–88% of PD patients have sleep problems that affect their daytime and nighttime functions (Factor, McAlarney, Sanchez‐Ramos, & Weiner, 1990; Goldman et al., 2013; Setthawatcharawanich, Limapichat, Sathirapanya, & Phabphal, 2014; Yu, Tan, & Wu, 2015). Although many studies performed in the past decade have investigated the clinical characteristics of sleep disturbance among patients with PD, some issues remain unclear and warrant further delineation. The most highlighted daytime sleep problem is excessive daytime sleepiness (EDS) (Salawu & Olokoba, 2015). However, whether EDS depends on nighttime problems remains uncertain. Some authors have argued that EDS could be an integral part of PD rather than the result of poor nighttime sleep quality (Goldman et al., 2013). In addition, whether clinical characteristics such as disease severity, Unified Parkinson's Disease Rating Scale (UPDRS) score, and the prescription of dopaminergic medication are risk factors for sleep disturbance remains controversial (Hoglund, Broman, Palhagen, Fredrikson, & Hagell, 2015; Kumar, Bhatia, & Behari, 2002; Ondo et al., 2001; Pandey, Bajaj, Wadhwa, & Anand, 2016; Svensson, Beiske, Loge, Beiske, & Sivertsen, 2012; Tholfsen, Larsen, Schulz, Tysnes, & Gjerstad, 2015).

Furthermore, few studies have focused on comparing the prevalence of sleep disturbance specifically in Asian and Caucasian populations. Intriguingly, although the reported percentages of poor sleepers in Asian patients are equal to or slightly higher than those among Western populations, the prevalence of EDS is lower in Asian than in Caucasian patients. In the Western countries, such as France, the Netherlands, Switzerland, Canada, and the United States, the percentage of poor sleepers ranged from 58.8% to 63% and EDS ranged from 40.6% to 57% (Brodsky, Godbold, Roth, & Olanow, 2003; Hobson et al., 2002; Louter, Munneke, Bloem, & Overeem, 2012; Poryazova, Benninger, Waldvogel, & Bassetti, 2010; Ratti et al., 2015; Zhu, van Hilten, & Marinus, 2016). In Asia, EDS was found to be considerably less prevalent among patients with PD. In China, Thailand, Singapore, and in the only report regarding Taiwan, the percentages of poor sleepers ranged from 37% to 64.5%, whereas EDS ranged from 15.1% to 32.3% (Chen et al., 2015; Setthawatcharawanich et al., 2014; Tan et al., 2002; Yu et al., 2015). From these perspectives, a larger scale survey is warranted to elucidate ethnic differences in sleep disturbances among patients with PD.

The aims of our study were to investigate the prevalence of daytime and nighttime sleep problems among Taiwanese patients with PD, to explore the predictive factors for sleep disturbance, and to correlate sleep disorders with clinical demographics and motor dysfunction among patients with PD.

## 2 MATERIAL AND METHODS

This was a cross‐sectional, questionnaire‐based interview investigation. Consecutive patients with PD were recruited from the neurology outpatient clinic of a tertiary medical center in Taiwan. All patients were Asians and satisfied the United Kingdom Parkinson's Disease Society Brain Bank Clinical Diagnostic Criteria. Patients with atypical parkinsonism, a history of brain surgery, or other psychiatric disorders were excluded. The Institutional Review Board of Chang Gung Memorial Hospital approved the study (approval number: 102‐4711C), and all patients provided informed consent.

We collected demographic data and medication prescribed for each patient on the day of the interview when they visited the outpatient clinic. The severity of PD was evaluated using the UPDRS and the Hoehn and Yahr stage (H&Y stage), and both scores were determined without stopping medication. Nighttime and daytime sleep problems were evaluated using the Pittsburgh Sleep Quality Index (PSQI) (Buysse, Reynolds, Monk, Berman, & Kupfer, 1989) and the Epworth Sleepiness Scale (ESS), respectively (Chen et al., 2002; Johns, 1991). We evaluated the sleep problems that might be relevant to PD symptoms by using the Parkinson's Disease Sleep Scale (PDSS) (Chaudhuri et al., 2002), and the quality of life of PD patients was assessed using the 39‐Item Parkinson's Disease Questionnaire (PDQ‐39) (Jenkinson, Fitzpatrick, Peto, Greenhall, & Hyman, 1997).

Following the generally accepted criteria, we use PSQI > 5 to identify PD patients as poor sleepers (Buysse et al., 1989) and ESS ≥ 10 to identify those with daytime sleep disorders (EDS) (Chen et al., 2002; Johns, 1991).

The PDSS is a 15‐item questionnaire validated in 2002 to identify nocturnal symptoms in patients with PD. The PDSS initially suggested eight categories to address the following domains: overall quality of night's sleep (item 1), sleep onset and maintenance insomnia (items 2 and 3), nocturnal restlessness (items 4 and 5), nocturnal psychosis (items 6 and 7), nocturia (items 8 and 9), nocturnal motor symptoms (items 10–13), sleep refreshment (item 14), and daytime dosing (item 15) (Table 1). With advancing understanding of nonmotor symptoms in patients with PD during the past decades, some items of the PDSS could be grouped together and represented by a single domain. For example, numbness (item 10) and pain (item 11) were originally grouped into nocturnal motor symptoms; however, these two items were actually nocturnal nonmotor symptoms of patients with PD. Nocturia (item 8) is autonomic manifestation of PD, and nocturnal restlessness (items 4 and 5) were manifestations of sleep disturbance among patients with PD. Both were closely related to the disease per se of PD. Some researchers also regrouped different items into special domains to explore aspects of nocturnal disturbance of specific interest (Ray Chaudhuri et al., 2012; Trenkwalder et al., 2011). Therefore, we categorized individual item into five domains to illustrate the different characteristics in the 15 items, and to correlate each domain with nighttime and daytime sleep problems (Table 1).

**Table 1:** The revised categories of PDSS items

Item	Question of PDSS	The original categories PDSS addressed	Revised categories of PDSS in this study
1	Overall quality of night's sleep	Overall quality of night sleep	Sleep quality (*The original issues could be lumped together*)
2	Having difficulty falling asleep	Sleep onset and maintenance insomnia	
3	Having difficulty staying asleep		
4	Restless of legs or arms at night	Nocturnal restless	Nocturnal PD symptoms (*The restless is usually caused by sleep problems relevant to PD itself. Dysautonomic symptoms, pain, and numbness are nonmotor symptoms of PD*)
5	Fidget in bed		
8	Get up at night to pass urine	Nocturia	
10	Numbness or tingling of arms or legs at night	Nocturnal motor symptoms	
11	Painful cramps in arms and legs while sleeping		
12	Early morning painful posturing of arms or legs		Nocturnal motor symptoms (*The item 9 evaluates the nocturnal urination specifically caused by motor off*)
13	On waking experiencing tremor		
9	Incontinence of urine due to “off” symptoms	Nocturia	
6	Distressing dreams of night	Nocturnal psychosis	Nocturnal psychosis
7	Distressing hallucination of night		
14	Tired and sleepy after waking in the morning	Sleep refreshment	Daytime sleepiness
15	Unexpectedly fallen asleep during the day	Daytime dozing	

PDSS, Parkinson's Disease Sleep Scale.

### 2.1 Statistical analysis

Statistical analysis of the data was performed using SPSS version 22.0. All clinical characteristics are presented as mean ± *SD*. Pearson's chi‐squared tests were performed to compare categorical variables, and independent sample *t* tests were used to compare continuous variables between two groups. H&Y stage of two groups was compared using Mann–Whitney *U* tests. Logistic regression analyses were conducted to determine the risk factors and predictors of sleep disturbance in PD patients. Statistical significance was set at *p* < .05.

## 3 RESULTS

A total of 225 patients completed the study. Table 2 shows their clinical demographics. Among the patients, 128 (56.9%) were male and 97 (43.1%) were female adults; the mean age of the patients was 65.7 ± 8.88 years. The mean age of symptom onset was 57.5 ± 9.9 years, the mean disease duration was 8.18 ± 5.20 years, and the percentage of patients in H&Y stage ≤1, 1.5, 2, 2.5, 3, and 4 were 22.7%, 14.7%, 25.3%, 14.7%, 20.4%, and 1.8%, respectively.

**Table 2:** Clinical data of 225 patients and the two comparing groups, “good sleepers vs. poor sleepers” and “patients with EDS vs. patients without EDS”

	All patients (*n* = 225)	Good sleepers (*n* = 104)	Poor sleepers (*n* = 121)	Without EDS (*n* = 165)	With EDS (*n* = 59)
Male (%)	128 (57)	62 (60)	66 (55)	88 (53)	39 (66)
Age (years)	65.7 ± 8.88	65.31 ± 9.03	66.04 ± 8.77	65.73 ± 9.08	65.68 ± 8.44
Age of onset (years)	57.53 ± 9.90	57.52 ± 10.25	57.55 ± 9.63	57.69 ± 10.10	57.05 ± 9.46
Disease duration (years)	8.18 ± 5.20	7.82 ± 4.80	8.50 ± 5.52	8.05 ± 5.15	8.63 ± 5.38
H&Y stage (%)			a		b a
Stage ≤1	51 (22.7)	26 (25)	25 (20.7)	43 (26.0)	8 (13.6)
Stage 1.5	33 (14.7)	18 (17.3)	15 (12.4)	26 (15.8)	7 (11.9)
Stage 2	57 (25.3)	28 (26.9)	29 (24.0)	38 (23.0)	19 (32.2)
Stage 2.5	33 (14.7)	14 (13.5)	19 (15.7)	26 (15.8)	7 (11.9)
Stage 3	46 (20.4)	17 (16.3)	29 (24.0)	28 (17.0)	17 (28.8)
Stage 4	4 (1.8)	1 (1)	3 (2.5)	3 (1.8)	1 (1.7)
UPDRS					
Total scores	36.08 ± 16.50	31.34 ± 14.24	40.18 ± 17.28^b^	128.34 ± 15.03	123.37 ± 17.32
Part I	2.99 ± 1.74	2.51 ± 1.51	3.41 ± 1.8^b^	2.88 ± 1.68	3.29 ± 1.89
Part II	8.56 ± 5.30	6.90 ± 4.34	10.00 ± 5.64^b^	8.48 ± 5.11	8.88 ± 5.82
Part III	22.77 ± 10.80	20.57 ± 10.17	24.68 ± 11.01^b^	22.45 ± 10.51	23.69 ± 11.70
LEDD (mg)	619.88 ± 389.33	568.40 ± 308.83	664.12 ± 443.63	612.23 ± 409.85	644.99 ± 329.84
Levodopa dosage (mg/day)	425.84 ± 324.92	368.47 ± 272.04	475.15 ± 358.11^b^	427.90 ± 339.07	423.93 ± 285.94
DA use (%)	162 (72)	82 (79)	80 (66)	112 (68)	49 (83)^c^
LED of DA (mg/day)	100.92 ± 92.80	111.95 ± 85.21	91.45 ± 98.22	93.42 ± 93.96	121.91 ± 87.67^c^
Hypnotic drug use (%)	142 (63)	56 (54)	86 (71)^b^	104 (63)	38 (64)
PSQI	6.95 ± 4.27	—	—	7.12 ± 4.39	6.51 ± 3.97
PDSS	127.08 ± 15.75	135.37 ± 9.41	119.95 ± 16.62^b^	128.34 ± 15.03	123.37 ± 17.32^c^
ESS	7.02 ± 4.62	7.28 ± 4.20	6.80 ± 4.95	—	—
PDQ‐39	20.11 ± 21.35	14.48 ± 16.09	25.02 ± 24.06^b^	19.58 ± 21.51	21.74 ± 21.16

DA, dopamine agonist; EDS, excessive daytime sleepiness; ESS, Epworth Sleepiness Scale; LEDD, levodopa equivalent daily dosage; LED, levodopa equivalent dosage; PDQ‐39, 39‐Item Parkinson's Disease Questionnaire; PDSS, Parkinson's Disease Sleep Scale; PSQI, Pittsburgh Sleep Quality Index; UPDRS, Unified Parkinson's Disease Rating Scale.

Data are presented as mean ± *SD*.

a Using Mann–Whitney *U* test.

b *p* < .05, comparison between good and poor sleepers.

c *p* < .05, comparison between patients with and without EDS.

**Figure 1:**
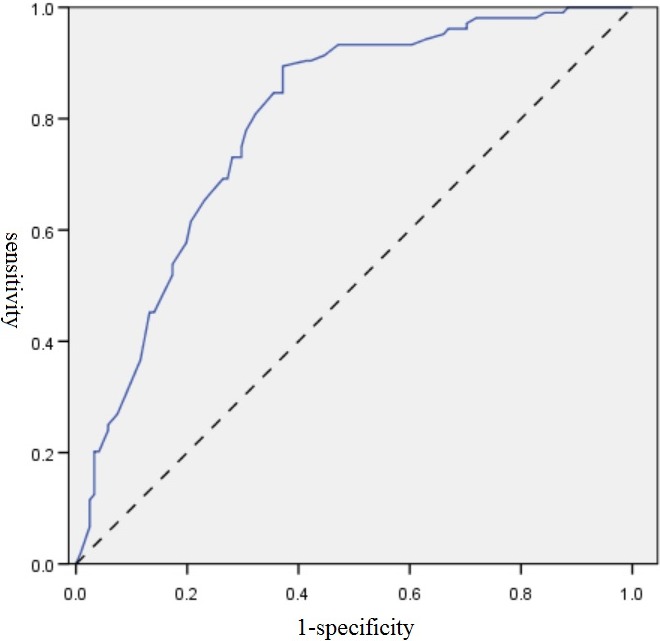
Receiver operating characteristic (ROC) curve for the Parkinson's Disease Sleep Scale (PDSS) score to predict poor sleepers among patients with PD. A PDSS score of <126 could predict poor sleep among patients with PD (<126.25, sensitivity 89.4%, 1‐specificity 37.2%)

**Figure 2:**
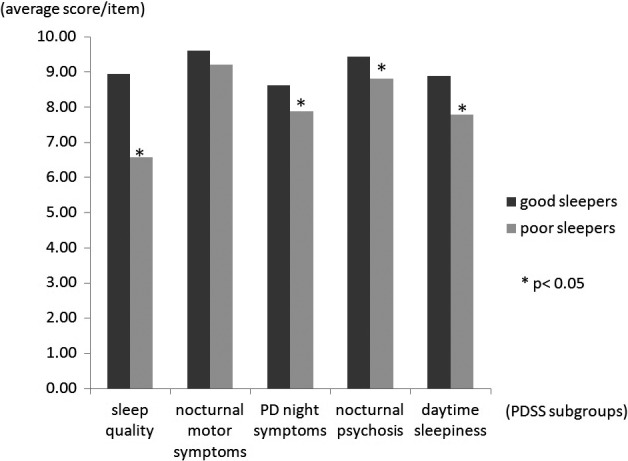
Comparing subgroups of Parkinson's Disease Sleep Scale (PDSS) of good and poor sleepers. The *y*‐axis represents the average score for each item in our subgrouping of PDSS. Compared with good sleepers, poor sleepers had significantly worse sleep quality (8.95 vs. 6.58, *p* < .001), nocturnal PD symptoms (8.61 vs. 7.88, *p* < .001), and psychosis (9.44 vs. 8.80, *p* < .001), and more daytime sleepiness (8.89 vs. 7.79, *p* = .001). No difference was observed in the nocturnal motor symptoms (9.60 vs. 9.2, *p* = .21)

Among the 225 recruited patients, 121 (53.8%) were defined as poor sleepers based on PSQI global scores of >5. Table 2 summarizes comparisons of the clinical characteristics of good and poor sleepers. Poor sleepers had significantly higher scores in the UPDRS total (*p* < .001), UPDRS part I (*p* < .001), UPDRS part II (*p* < .001), UPDRS part III (*p* = .004), and PDQ‐39 (*p* < .001), lower scores in the PDSS (*p* < .001), and more levodopa daily dosage (*p* = .012). The percentage of patients using hypnotic drugs was higher in poor sleepers (*p* = .008). Logistic regression was used to correlate and predict the risk factors of poor sleep among patients with PD. Variables with *p* < .05 were selected, which included UPDRS total score, PDSS, PDQ‐39, levodopa daily dosage, and hypnotic drug use. Of the studied variables, PDSS total score had the strongest effect on poor sleepers (*p* < .001, OR = 0.909). The receiver operating characteristic (ROC) curve indicated that a PDSS score of <126 could predict poor sleep among patients with PD (<126.25, sensitivity 89.4%, 1‐specificity 37.2%; Figure 1). Further analysis of the average score for each item in our subgrouping of PDSS showed that poor sleepers had lower scores for sleep quality (8.95 ± 1.16 vs. 6.58 ± 2.50, *p* < .001), nighttime PD symptoms (8.61 ± 0.96 vs. 7.88 ± 1.44, *p* < .001), nocturnal psychosis (9.44 ± 1.01 vs. 8.80 ± 1.74, *p* < .001), and daytime sleepiness (8.89 ± 2.00 vs. 7.79 ± 2.91, *p* = .001). However, no difference was observed in the nocturnal motor symptoms of good and poor sleepers (9.60 ± 1.12 vs. 9.2 ± 1.45, *p* = .21; Figure 2).

**Figure 3:**
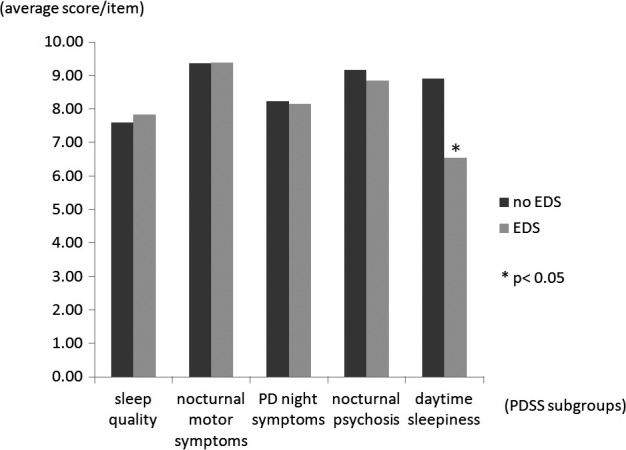
Comparing subgroups of Parkinson's Disease Sleep Scale (PDSS) of PD patients with and without excessive daytime sleepiness (EDS). The *y*‐axis represents the average score for each item in our subgrouping of PDSS. Compared with patients without EDS, patients with EDS had more daytime sleepiness (8.91 vs. 6.55, *p* < .001), and this subgroup was essentially the same parameters like Epworth Sleepiness Scale (ESS). It is worthy to note that except this domain, no difference was observed in the sleep quality (7.61 vs. 7.84, *p* = .50), nocturnal motor symptoms (9.38 vs. 9.40, *p* = .92), nocturnal PD symptoms (8.24 vs. 8.17, *p* = .70), and psychosis (9.18 vs. 8.85, *p* = .15)

Epworth Sleepiness Scale data were missing for one patient; 59 patients (26.3%) had EDS. Comparing patients with and without EDS revealed that EDS was associated with more advanced H&Y stage (*p* = .025), lower PDSS score (123.37 ± 17.32 vs. 128.34 ± 15.03, *p* = .038), more frequent use of dopamine agonists (DAs) (68% vs. 83%, *p* = .034), and more levodopa equivalent dosage of DA (LED of DA) (121.91 ± 87.67 vs. 93.42 ± 93.96, *p* = .043). However, no differences were observed in the levodopa equivalent daily dosage (LEDD) (*p* = .58) and the item of prescribed dopaminergic medication between EDS and non‐EDS groups (Table 4). Sixty‐three percent of patients in our cohort used hypnotic drugs which included benzodiazepines, Z drugs (zolpidem, zopiclone, and zaleplon), antidepressants, and neuroleptics. No differences were found in the ratio of hypnotic drug use (*p* = .851) and each category of hypnotic drug between patients with and without EDS (Table 4). We found no significant difference among the subgroups of PDSS, except with the daytime sleepiness subgroup (8.91 ± 1.81 vs. 6.55 ± 3.50, *p* < .001; Figure 3).

## 4 DISCUSSION

The sleep profiles of 225 consecutive Taiwanese patients with PD in a single medical center were documented. This series had four major findings: (1) 53.8% of patients were poor sleepers, and 26.3% had EDS. (2) The poor sleepers had higher scores in the UPDRS and PDQ‐39, lower scores in the PDSS, more levodopa dosage, as well as more severe nocturnal PD symptoms and psychosis. In addition, the percentage of PD patients receiving hypnotics was higher in poor sleepers than in good sleepers. (3) EDS was positively correlated with advanced H&Y stage, DA use, and LED of DA, but not with LEDD. Furthermore, hypnotic drugs did not increase the occurrence of EDS. (4) A PDSS total score of <126 was discovered to predict poor sleep among patients with PD.

To the best of our knowledge, this series was the second study of the prevalence of sleep disturbance among Taiwanese patients with PD. Overall, 53.8% of patients were defined as poor sleepers, which was in agreement with the other study of Taiwanese patients by Yu et al. (2015) and similar to Asia and Western series that used the PSQI (Table 3). Although a control group was not recruited in our cohort, another general study of 760 representative Taiwanese individuals between 2010 and 2013 showed that 46.6% were poor sleepers, which is lower than the result obtained from our data (Tai, Wang, & Yang, 2015). Our findings further highlight the importance of increasing the awareness of sleep problems in PD patients.

**Table 3:** Prevalence of poor sleep and EDS in Western and Asian countries

	Poor sleepers (PSQI > 5) (%)	EDS (%)
USA, Goldman et al.	59.1	49.5
France, Ratti et al.	63	—
Netherlands, Louter et al.	58.8	—
Netherlands, Zhu et al.	—	43
Switzerland, Poryazova et al.	—	57
Canada, Hobson et al.	—	51
USA, Brodsky et al.	—	40.6
Taiwan, Yu et al.	64.4	23.8
China, Chen et al.	64.5	32.3
Thailand, Setthawatcharawanich et al.	37	15.1
Singapore, Tan et al.	—	19.9
Taiwan (our study)	53.8	26.3

EDS, excessive daytime sleepiness; PSQI, Pittsburgh Sleep Quality Index.

Both studies performed in Taiwan documented low prevalence of EDS among patients with PD: 26.3% in our study and 23.8% in the previous study (Yu et al., 2015). The occurrence of EDS among patients with PD was lower in Asian than in Caucasian populations in the literature. Studies from Asia using the ESS have reported that the prevalence of EDS ranged from 15.1% to 32.3%; however, prevalence ranging from 40.6% to 57% was reported for Europe and North America (Brodsky et al., 2003; Chen et al., 2015; Goldman et al., 2013; Hobson et al., 2002; Louter et al., 2012; Poryazova et al., 2010; Ratti et al., 2015; Setthawatcharawanich et al., 2014; Tan et al., 2002; Yu et al., 2015; Zhu et al., 2016) (Table 3). Disease severity, disease duration, dopaminergic therapy, and hypnotic medication have been suggested to contribute to EDS; however, neither of them could fully explain this discrepancy. The disease severity of the patients were similar in these Western and Asian studies, with H&Y stages between 2 and 3 (Poryazova et al., 2010; Setthawatcharawanich et al., 2014; Tan et al., 2002; Yu et al., 2015; Zhu et al., 2016). In a study of 638 Canadian patients with PD with H&Y stage of 2.2 ± 0.68, Hobson et al. (2002) reported that 51% had EDS; however, in our study, only 26.3% of patients with a similar H&Y stage had EDS. In our patient cohort and that investigated in Switzerland by Poryazova et al., the mean age (65.7 ± 8.88 vs. 65 ± 10 years) and disease duration (8.2 ± 5.2 vs. 8.2 ± 6.6 years) were similar; however, 57% of the patients studied by Poryazova et al. (2010) had EDS. In a study of 126 patients with PD in Cambridgeshire (UK), 49% had EDS even though the mean disease duration was only 3.54 years (Breen, Williams‐Gray, Mason, Foltynie, & Barker, 2013).

Medication was an important factor contributing to EDS, and might influence prevalence of EDS. In our series, no differences were observed in the category and dosage of prescribed hypnotics between the EDS and non‐EDS groups. The percentages of PD patients receiving benzodiazepines were no difference between the EDS and non‐EDS groups in our patient cohort as well as those identified by Zhu et al. (2016) in the Netherland population; however, 43% of patients studied by Zhu et al. had EDS. The ratio of DA utilization in our patients was higher than that reported in the United States (Crispo et al., 2015). More than 50% of the patients in our series received DAs, which may increase daytime sleepiness. However, the prevalence of EDS among our patients was even lower. Given these findings, ethnicity‐specific determinants and genetic predisposition should play some roles in the discrepancy. Some authors have disclosed that patients with the catechol‐O‐methyltransferase (COMT) Met/Met genotype tend to have higher ESS scores (Frauscher et al., 2004). However, the genotype distribution of COMT Val158Met polymorphism in Caucasians quite differs from that observed in Northeastern Asians (the Val/Val genotype frequencies are 25% and 10%, respectively) (Lachman et al., 1996).

Despite the prevalence of EDS is relatively low among Taiwanese patients with PD, it is an important issue to identify the risk factors of EDS. In our series, H&Y stage, DA utilization, and DA dosage were the only three variables identified as differing significantly between EDS and non‐EDS patients. Through a review of recent articles, Arnulf (2005) summarized that EDS is associated with more advanced disease, higher doses of levodopa‐equivalent, and DAs use. The levodopa may have a soporific effect and enhance the somnolence with increasing dosage (O'Suilleabhain & Dewey, 2002). However, in our study, a significant correlation was not identified between levodopa dosage and EDS (Table 2), and two other studies also reported no such correlation (Gjerstad, Alves, Wentzel‐Larsen, Aarsland, & Larsen, 2006; Kumar, Bhatia, & Behari, 2003). DAs therapy contributes to EDS and sudden somnolence (Hauser, Gauger, Anderson, & Zesiewicz, 2000; Pal, Bhattacharya, Agapito, & Chaudhuri, 2001). Our results confirmed that EDS could be augmented by more DA use and higher DA dosage (Table 2). Although DA utilization is a risk factor of EDS, we could not identify different impact on daytime sleepiness of different categories of DAs in our patient cohorts. Neither the biochemical characteristics (ergot DAs vs. nonergot DAs) nor the receptors profile (D2 receptors preferential vs. D3 receptors preferential) made the EDS prevalence different between PD patients with and without EDS (Table 4). In spite of our findings were in concord with previous reports (Hauser et al., 2000; Pal et al., 2001), the issues of drugs related EDS warrant further in‐depth analysis with large sample and well‐designed study. It is believed that hypnotics may increase EDS, and medications like amantadine, rasagiline, and selegiline may decrease EDS through the effects of their stimulating metabolite, nevertheless, our analysis could not support the general assumption (Table 4). Benzodiazepines were currently reported to be associated with lower score in SCOPA‐SLEEP‐Daytime Sleepiness (Zhu et al., 2016). A case–control study described that clonazepam, the most prescribed benzodiazepines in our study, decreased the occurrence of EDS among patients with PD (Shpirer et al., 2006). Except for H&Y stage and DA utilization, we found occurrence of the EDS was not correlated with the UPDRS (disease severity), the PDSS (nocturnal PD symptoms), and the PSQI (Figure 3). Our results supported the notion that EDS represents a separate manifestation of PD and that it is merely dependent on disease rather than on nocturnal disturbance and nighttime sleep problems (Goldman et al., 2013; Suzuki et al., 2008).

**Table 4:** Numbers of the user of dopaminergic agents and hypnotic drugs prescribed among patients with and without EDS

Medication	User (*N*)	With EDS (*N*)	Without EDS (*N*)	*p* value
Pergolide	2	0	2	.396
Bromocriptine	1	0	1	.549
Pramipexole	101	33	68	.051
Ropinirole	58	14	44	.658
Rotigotine	5	2	3	.483
Rasagiline	5	1	4	.745
Selegiline	55	11	44	.219
Amantadine	73	22	51	.370
Benzodiazepines	139	36	103	.848
Z drugs^a^	10	3	7	.788
Antidepressants	24	6	18	.875
Neuroleptics	8	3	5	.466

EDS, excessive daytime sleepiness.

a Z drugs includes zolpidem, zopiclone, and zaleplon.

Aside from the daytime problem of EDS in patients with PD, nighttime sleep problems also significantly impair the quality of life in PD patients. Identifying risk factors that predict nighttime dysfunction are important for clinical practice. We clarified disease severity (identified by UPDRS), nocturnal PD symptom (identified by PDSS), quality of life (identified by PDQ‐39), and medications (levodopa and hypnotics) were correlated with poor sleep in our cohort (Table 2 and Figure 2). We found that the poor sleepers had significant higher UPDRS scores. Our finding contrasted with a community‐based study in Norway (Svensson et al., 2012), but is in agreement with a case–control study conducted in India (Kumar et al., 2002). In addition, poor sleep was positively correlated with the dosage of levodopa and hypnotics in our and two other studies (Antczak et al., 2013; Verbaan, van Rooden, Visser, Marinus, & van Hilten, 2008). Levodopa can improve sleep efficiency with reduced sleep latency by improving motor scores (Askenasy & Yahr, 1985; Ferreira, Prabhakar, & Kharbanda, 2014; Kales, Ansel, Markham, Scharf, & Tan, 1971). Therefore, instead of higher dosage of levodopa leading to poor sleep, it is possible that complaint of poor sleep made poor sleepers in our cohort receive higher dosage of levodopa than good sleepers. We also discovered that the PDSS had a strong effect on and is a useful predictor of poor sleep among patients with PD. Some researchers have also tried to analyze the correlation of PDSS items and subgroup with poor sleep. However, these reports have revealed heterogeneous results (Uemura et al., 2009; Yu et al., 2015). Using ROC curve approach, we suggested that a PDSS total score <126 might be a reliable threshold to indicate poor sleep among patients with PD.

### 4.1 Limitation

Our study was designed as a questionnaire‐based interview investigation. Lacking of strong objective data such as polysomnography or mean sleep latency tests limited the findings. In addition, patients needed enough mental and physical condition to complete the questionnaires. Therefore, the severity of PD of most patients was not advanced.

## CONFLICT OF INTEREST

The authors declare no financial or other conflicts of interest.

## References

[brb3806-bib-0001] Antczak, J. M. , Rakowicz, M. J. , Banach, M. , Derejko, M. , Sienkiewicz, J. , Zalewska, U. , … Jernajczyk, W. (2013). Negative influence of L‐dopa on subjectively assessed sleep but not on nocturnal polysomnography in Parkinson's disease. Pharmacological Reports, 65(3), 614–623. 23950583 10.1016/s1734-1140(13)71038-7

[brb3806-bib-0002] Arnulf, I. (2005). Excessive daytime sleepiness in parkinsonism. Sleep Medicine Reviews, 9(3), 185–200. 15893249 10.1016/j.smrv.2005.01.001

[brb3806-bib-0101] Askenasy, J. J. , & Yahr, M. D. (1985). Reversal of sleep disturbance in Parkinson's disease by antiparkinsonian therapy: a preliminary study. Neurology, 35(4), 527–532. 3982638 10.1212/wnl.35.4.527

[brb3806-bib-0003] Breen, D. P. , Williams‐Gray, C. H. , Mason, S. L. , Foltynie, T. , & Barker, R. A. (2013). Excessive daytime sleepiness and its risk factors in incident Parkinson's disease. Journal of Neurology, Neurosurgery, and Psychiatry, 84(2), 233–234. 10.1136/jnnp-2012-304097 23184153

[brb3806-bib-0004] Brodsky, M. A. , Godbold, J. , Roth, T. , & Olanow, C. W. (2003). Sleepiness in Parkinson's disease: A controlled study. Movement Disorders, 18(6), 668–672. 12784270 10.1002/mds.10429

[brb3806-bib-0005] Buysse, D. J. , Reynolds, C. F. 3rd , Monk, T. H. , Berman, S. R. , & Kupfer, D. J. (1989). The Pittsburgh sleep quality index: A new instrument for psychiatric practice and research. Psychiatry Research, 28(2), 193–213. 2748771 10.1016/0165-1781(89)90047-4

[brb3806-bib-0006] Chaudhuri, K. R. , Pal, S. , DiMarco, A. , Whately‐Smith, C. , Bridgman, K. , Mathew, R. , … Trenkwalder, C. (2002). The Parkinson's disease sleep scale: A new instrument for assessing sleep and nocturnal disability in Parkinson's disease. Journal of Neurology, Neurosurgery, and Psychiatry, 73(6), 629–635. 10.1136/jnnp.73.6.629 PMC1757333 12438461

[brb3806-bib-0007] Chen, N. H. , Johns, M. W. , Li, H. Y. , Chu, C. C. , Liang, S. C. , Shu, Y. H. , … Wang, P. C. (2002). Validation of a Chinese version of the Epworth sleepiness scale. Quality of Life Research, 11(8), 817–821. 12482165 10.1023/a:1020818417949

[brb3806-bib-0008] Chen, J. , Yao, J. , Chen, L. , Miao, H. , Mao, C. , & Liu, C. (2015). Sleep disorders associated with essential tremor and Parkinson's disease. Zhonghua Yi Xue Za Zhi, 95(3), 205–209. 25877032

[brb3806-bib-0009] Crispo, J. A. , Fortin, Y. , Thibault, D. P. , Emons, M. , Bjerre, L. M. , Kohen, D. E. , … Krewski, D. (2015). Trends in inpatient antiparkinson drug use in the USA, 2001‐2012. European Journal of Clinical Pharmacology, 71(8), 1011–1019. 26081062 10.1007/s00228-015-1881-4 PMC4500853

[brb3806-bib-0010] Factor, S. A. , McAlarney, T. , Sanchez‐Ramos, J. R. , & Weiner, W. J. (1990). Sleep disorders and sleep effect in Parkinson's disease. Movement Disorders, 5(4), 280–285. 2259351 10.1002/mds.870050404

[brb3806-bib-0102] Ferreira, T. , Prabhakar, S. , & Kharbanda, P. S. (2014). Sleep disturbances in drug naive Parkinson's disease (PD) patients and effect of levodopa on sleep. Annals of Indian Academy of Neurology, 17(4), 416–419. 25506163 10.4103/0972-2327.144016 PMC4251015

[brb3806-bib-0011] Frauscher, B. , Hogl, B. , Maret, S. , Wolf, E. , Brandauer, E. , Wenning, G. K. , … Poewe, W. (2004). Association of daytime sleepiness with COMT polymorphism in patients with Parkinson's disease: A pilot study. Sleep, 27(4), 733–736. 15283009 10.1093/sleep/27.4.733

[brb3806-bib-0103] Gjerstad, M. D. , Alves, G. , Wentzel‐Larsen, T. , Aarsland, D. , & Larsen, J. P. (2006). Excessive daytime sleepiness in Parkinson disease: is it the drugs or the disease? Neurology, 67(5), 853–858. 16966550 10.1212/01.wnl.0000233980.25978.9d

[brb3806-bib-0012] Goldman, J. G. , Ghode, R. A. , Ouyang, B. , Bernard, B. , Goetz, C. G. , & Stebbins, G. T. (2013). Dissociations among daytime sleepiness, nighttime sleep, and cognitive status in Parkinson's disease. Parkinsonism & Related Disorders, 19(9), 806–811. 23735187 10.1016/j.parkreldis.2013.05.006 PMC3729741

[brb3806-bib-0013] Hauser, R. A. , Gauger, L. , Anderson, W. M. , & Zesiewicz, T. A. (2000). Pramipexole‐induced somnolence and episodes of daytime sleep. Movement Disorders, 15(4), 658–663. 10928575 10.1002/1531-8257(200007)15:4<658::aid-mds1009>3.0.co;2-n

[brb3806-bib-0014] Hobson, D. E. , Lang, A. E. , Martin, W. R. , Razmy, A. , Rivest, J. , & Fleming, J. (2002). Excessive daytime sleepiness and sudden‐onset sleep in Parkinson disease: A survey by the Canadian Movement Disorders Group. JAMA, 287(4), 455–463. 11798367 10.1001/jama.287.4.455

[brb3806-bib-0015] Hoglund, A. , Broman, J. E. , Palhagen, S. , Fredrikson, S. , & Hagell, P. (2015). Is excessive daytime sleepiness a separate manifestation in Parkinson's disease? Acta Neurologica Scandinavica, 132(2), 97–104. 25630925 10.1111/ane.12378

[brb3806-bib-0016] Jenkinson, C. , Fitzpatrick, R. , Peto, V. , Greenhall, R. , & Hyman, N. (1997). The Parkinson's Disease Questionnaire (PDQ‐39): Development and validation of a Parkinson's disease summary index score. Age and Ageing, 26(5), 353–357. 9351479 10.1093/ageing/26.5.353

[brb3806-bib-0017] Johns, M. W. (1991). A new method for measuring daytime sleepiness: The Epworth sleepiness scale. Sleep, 14(6), 540–545. 1798888 10.1093/sleep/14.6.540

[brb3806-bib-0104] Kales, A. , Ansel, R. D. , Markham, C. H. , Scharf, M. B. , & Tan, T. L. (1971). Sleep in patients with Parkinson's disease and normal subjects prior to and following levodopa administration. Clinical Pharmacology & Therapeutics, 12(2), 397–406. 4325251 10.1002/cpt1971122part2397

[brb3806-bib-0018] Kumar, S. , Bhatia, M. , & Behari, M. (2002). Sleep disorders in Parkinson's disease. Movement Disorders, 17(4), 775–781. 12210875 10.1002/mds.10167

[brb3806-bib-0105] Kumar, S. , Bhatia, M. , & Behari, M. (2003). Excessive daytime sleepiness in Parkinson's disease as assessed by Epworth Sleepiness Scale (ESS). Sleep Medicine, 4(4), 339–342. 14592307 10.1016/s1389-9457(03)00105-9

[brb3806-bib-0019] Lachman, H. M. , Papolos, D. F. , Saito, T. , Yu, Y. M. , Szumlanski, C. L. , & Weinshilboum, R. M. (1996). Human catechol‐O‐methyltransferase pharmacogenetics: Description of a functional polymorphism and its potential application to neuropsychiatric disorders. Pharmacogenetics, 6(3), 243–250. 8807664 10.1097/00008571-199606000-00007

[brb3806-bib-0020] Louter, M. , Munneke, M. , Bloem, B. R. , & Overeem, S. (2012). Nocturnal hypokinesia and sleep quality in Parkinson's disease. Journal of the American Geriatrics Society, 60(6), 1104–1108. 22642534 10.1111/j.1532-5415.2012.03966.x

[brb3806-bib-0021] Ondo, W. G. , Dat Vuong, K. , Khan, H. , Atassi, F. , Kwak, C. , & Jankovic, J. (2001). Daytime sleepiness and other sleep disorders in Parkinson's disease. Neurology, 57(8), 1392–1396. 11673578 10.1212/wnl.57.8.1392

[brb3806-bib-0022] O'Suilleabhain, P. E. , & Dewey, R. B. Jr (2002). Contributions of dopaminergic drugs and disease severity to daytime sleepiness in Parkinson disease. Archives of Neurology, 59(6), 986–989. 12056935 10.1001/archneur.59.6.986

[brb3806-bib-0023] Pal, S. , Bhattacharya, K. F. , Agapito, C. , & Chaudhuri, K. R. (2001). A study of excessive daytime sleepiness and its clinical significance in three groups of Parkinson's disease patients taking pramipexole, cabergoline and levodopa mono and combination therapy. Journal of Neural Transmission (Vienna), 108(1), 71–77. 10.1007/s007020170098 11261748

[brb3806-bib-0024] Pandey, S. , Bajaj, B. K. , Wadhwa, A. , & Anand, K. S. (2016). Impact of sleep quality on the quality of life of patients with Parkinson's disease: A questionnaire based study. Clinical Neurology and Neurosurgery, 148, 29–34. 27372436 10.1016/j.clineuro.2016.06.014

[brb3806-bib-0025] Poryazova, R. , Benninger, D. , Waldvogel, D. , & Bassetti, C. L. (2010). Excessive daytime sleepiness in Parkinson's disease: Characteristics and determinants. European Neurology, 63(3), 129–135. 20090346 10.1159/000276402

[brb3806-bib-0026] Ratti, P. L. , Negre‐Pages, L. , Perez‐Lloret, S. , Manni, R. , Damier, P. , Tison, F. , … Rascol, O. (2015). Subjective sleep dysfunction and insomnia symptoms in Parkinson's disease: Insights from a cross‐sectional evaluation of the French CoPark cohort. Parkinsonism & Related Disorders, 21(11), 1323–1329. 26411501 10.1016/j.parkreldis.2015.09.025

[brb3806-bib-0027] Ray Chaudhuri, K. , Martinez‐Martin, P. , Rolfe, K. A. , Cooper, J. , Rockett, C. B. , Giorgi, L. , & Ondo, W. G. (2012). Improvements in nocturnal symptoms with ropinirole prolonged release in patients with advanced Parkinson's disease. European Journal of Neurology, 19(1), 105–113. 21699627 10.1111/j.1468-1331.2011.03442.x

[brb3806-bib-0028] Salawu, F. , & Olokoba, A. (2015). Excessive daytime sleepiness and unintended sleep episodes associated with Parkinson's disease. Oman Medical Journal, 30(1), 3–10. 25829994 10.5001/omj.2015.02 PMC4371466

[brb3806-bib-0029] Setthawatcharawanich, S. , Limapichat, K. , Sathirapanya, P. , & Phabphal, K. (2014). Excessive daytime sleepiness and nighttime sleep quality in Thai patients with Parkinson's disease. Journal of the Medical Association of Thailand, 97(10), 1022–1027. 25632617

[brb3806-bib-0030] Shpirer, I. , Miniovitz, A. , Klein, C. , Goldstein, R. , Prokhorov, T. , Theitler, J. , … Rabey, J. M. (2006). Excessive daytime sleepiness in patients with Parkinson's disease: A polysomnography study. Movement Disorders, 21(9), 1432–1438. 16773617 10.1002/mds.21002

[brb3806-bib-0031] Suzuki, K. , Miyamoto, T. , Miyamoto, M. , Okuma, Y. , Hattori, N. , Kamei, S. , … Hirata, K. (2008). Excessive daytime sleepiness and sleep episodes in Japanese patients with Parkinson's disease. Journal of the Neurological Sciences, 271(1–2), 47–52. 18436241 10.1016/j.jns.2008.03.008

[brb3806-bib-0032] Svensson, E. , Beiske, A. G. , Loge, J. H. , Beiske, K. K. , & Sivertsen, B. (2012). Sleep problems in Parkinson's disease: A community‐based study in Norway. BMC Neurology, 12, 71. 22883600 10.1186/1471-2377-12-71 PMC3472275

[brb3806-bib-0033] Tai, S. Y. , Wang, W. F. , & Yang, Y. H. (2015). Current status of sleep quality in Taiwan: A nationwide walk‐in survey. Annals of General Psychiatry, 14, 36. 26535047 10.1186/s12991-015-0078-7 PMC4630925

[brb3806-bib-0034] Tan, E. K. , Lum, S. Y. , Fook‐Chong, S. M. , Teoh, M. L. , Yih, Y. , Tan, L. , … Wong, M. C. (2002). Evaluation of somnolence in Parkinson's disease: Comparison with age‐ and sex‐matched controls. Neurology, 58(3), 465–468. 11839852 10.1212/wnl.58.3.465

[brb3806-bib-0106] Trenkwalder, C. , Kies, B. , Rudzinska, M. , Fine, J. , Nikl, J. , Honczarenko, K. , … Chaudhuri, K. R. (2011). Rotigotine effects on early morning motor function and sleep in Parkinson's disease: a double‐blind, randomized, placebo‐controlled study (RECOVER). Movement Disorders, 26(1), 90–99. 21322021 10.1002/mds.23441 PMC3072524

[brb3806-bib-0035] Tholfsen, L. K. , Larsen, J. P. , Schulz, J. , Tysnes, O. B. , & Gjerstad, M. D. (2015). Development of excessive daytime sleepiness in early Parkinson disease. Neurology, 85(2), 162–168. 26085603 10.1212/WNL.0000000000001737

[brb3806-bib-0036] Uemura, Y. , Nomura, T. , Inoue, Y. , Yamawaki, M. , Yasui, K. , & Nakashima, K. (2009). Validation of the Parkinson's disease sleep scale in Japanese patients: A comparison study using the Pittsburgh sleep quality index, the Epworth sleepiness scale and polysomnography. Journal of the Neurological Sciences, 287(1–2), 36–40. 19804890 10.1016/j.jns.2009.09.015

[brb3806-bib-0037] Verbaan, D. , van Rooden, S. M. , Visser, M. , Marinus, J. , & van Hilten, J. J. (2008). Nighttime sleep problems and daytime sleepiness in Parkinson's disease. Movement Disorders, 23(1), 35–41. 17960797 10.1002/mds.21727

[brb3806-bib-0038] Yu, R. L. , Tan, C. H. , & Wu, R. M. (2015). The impact of nocturnal disturbances on daily quality of life in patients with Parkinson's disease. Neuropsychiatric Disease and Treatment, 11, 2005–2012. 26273203 10.2147/NDT.S85483 PMC4532217

[brb3806-bib-0039] Zhu, K. , van Hilten, J. J. , & Marinus, J. (2016). Course and risk factors for excessive daytime sleepiness in Parkinson's disease. Parkinsonism & Related Disorders, 24, 34–40. 26846609 10.1016/j.parkreldis.2016.01.020

